# P-777. Difference in Clinically Ready For Discharge Status and Actual Discharge Date in Hospitalized Complicated Urinary Tract Infection Patients Treated with Intravenous Carbapenem

**DOI:** 10.1093/ofid/ofaf695.988

**Published:** 2026-01-11

**Authors:** Serena P Koenig, Jeffrey J Ellis, Douglas Boettner, Lindsey Parker, Rose Chang, Louise Yu, Emily Reichert, Joanne Chukwueke, Zheyi Cao, Yichuan Grace Hseih, Christopher Herrick, Mei Sheng Duh, Shawn N Murphy

**Affiliations:** Brigham and Women's Hospital, Boston, MA, United States; Harvard Medical School, Boston, MA, United States, Boston, MA; GSK, Collegeville, Pennsylvania; GSK, Collegeville, PA, United States, Collegeville, Pennsylvania; GSK, Collegeville, PA, United States, Collegeville, Pennsylvania; Analysis Group, Inc., Boston, Massachusetts; Analysis Group Inc., Boston, MA, United States, Boston, Massachusetts; Analysis Group, Inc., Boston, MA, United States, Boston, Massachusetts; Analysis Group, Inc., Boston, MA, United States, Boston, Massachusetts; Analysis Group, Inc., Boston, MA, United States, Boston, Massachusetts; Harvard Medical School, Boston, MA, United States; Mass General Brigham, Somerville, MA, United States, Boston, Massachusetts; Mass General Brigham, Somerville, MA, United States, Somerville, Massachusetts; Analysis Group Inc., Boston, MA, United States, Boston, Massachusetts; Harvard Medical School, Boston, MA, United States; Mass General Brigham, Somerville, MA, United States; Massachusetts General Hospital, Boston, MA, United States, Boston, Massachusetts

## Abstract

**Background:**

Patients hospitalized with complicated urinary tract infections (cUTI) caused by multidrug-resistant uropathogens often need intravenous (IV) carbapenems, which can prolong inpatient (IP) stay and lead to increased adverse outcomes and resource utilization. This study aimed to determine the difference in duration between clinically ready for discharge (CRFD) status and actual hospital discharge date for hospitalized patients with cUTI treated with IV carbapenems.

**Figure:**
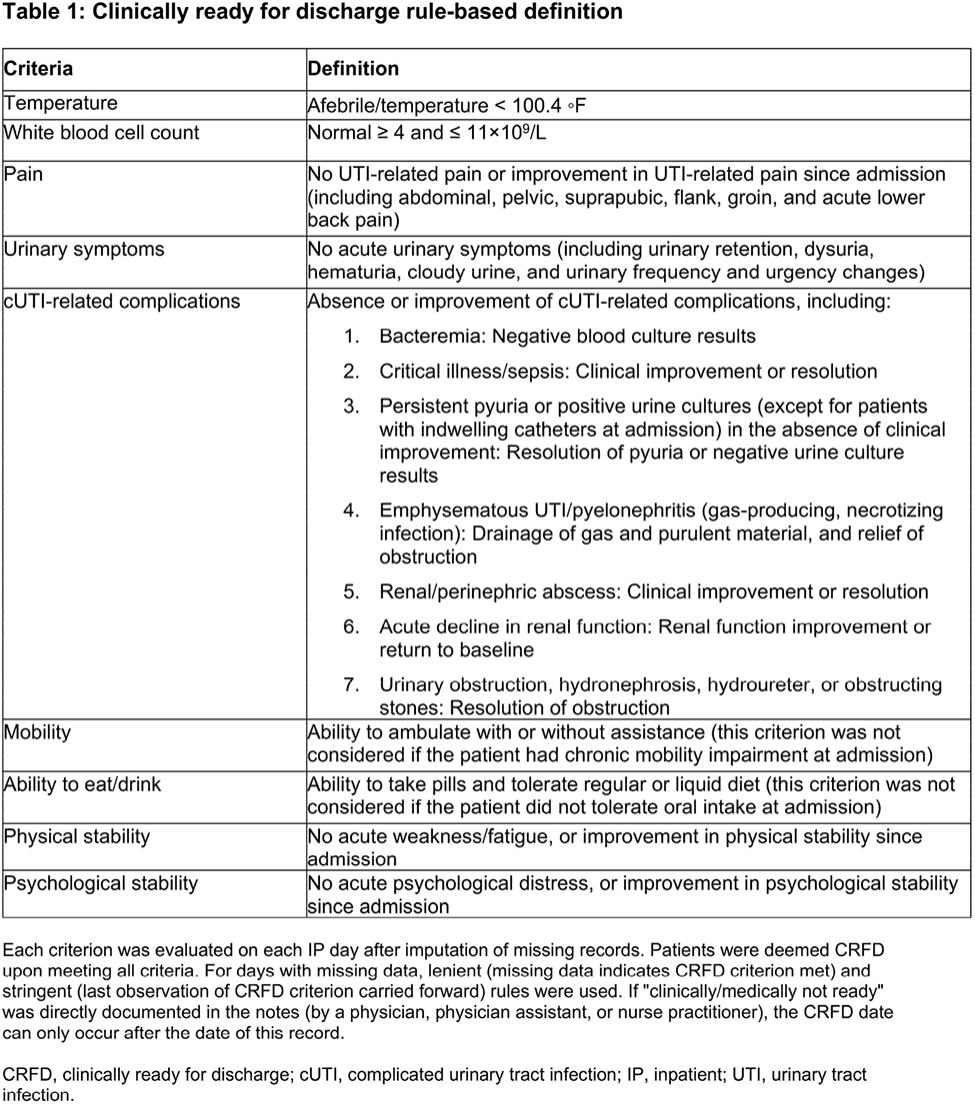

**Figure:**
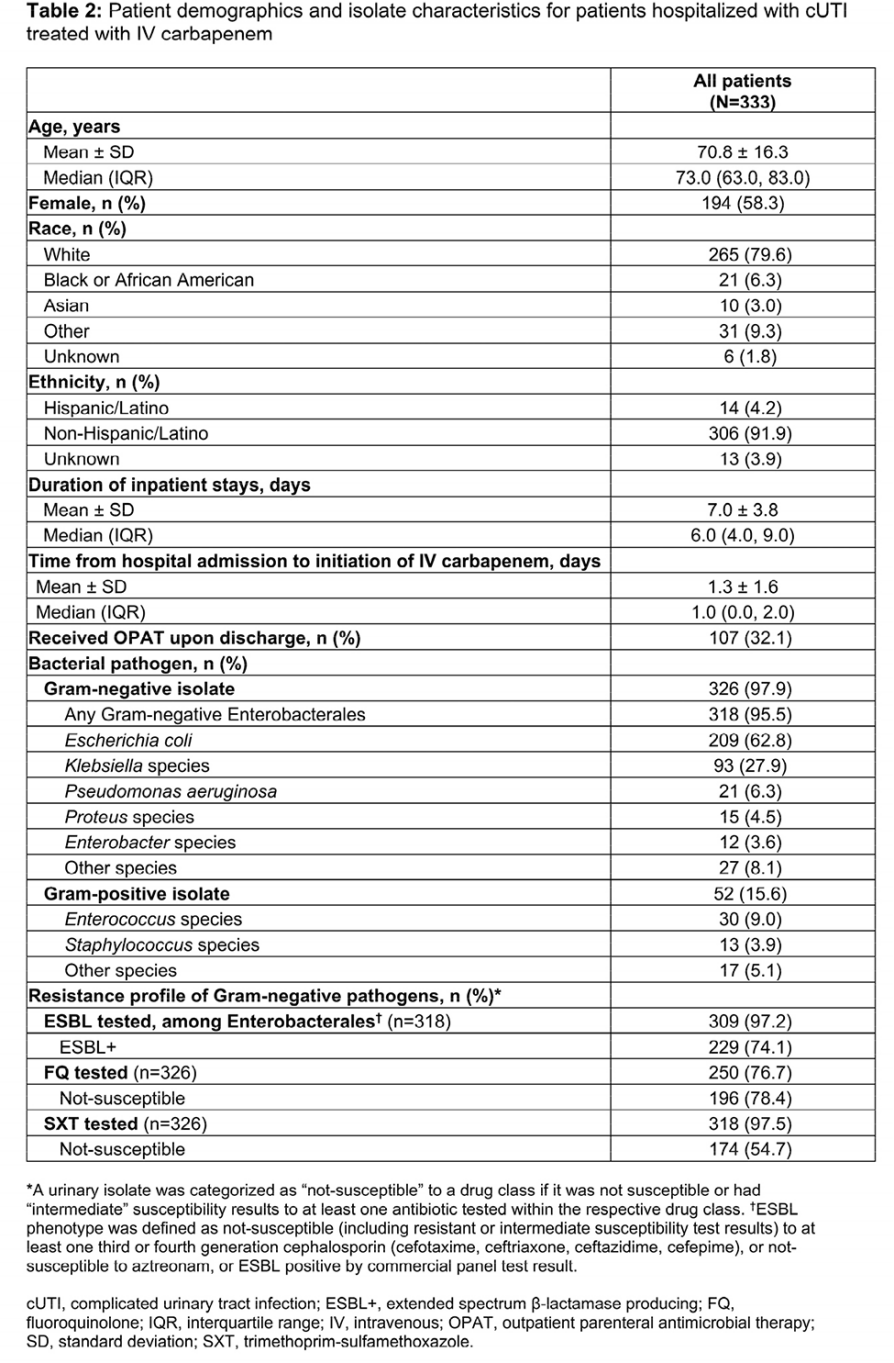

**Methods:**

Patients aged ≥18 years who received IV carbapenem for cUTI with a positive urine culture between January 2019 –June 2024 were assessed retrospectively using structured electronic health records and unstructured clinical notes from the Mass General Brigham Research Patient Data Registry. Stringent (missing data based on the last observation of CRFD criterion carried forward) and lenient (missing data assumed to indicate CRFD criterion met) rule-based definitions for CRFD (Table 1), based on literature review and clinical guidelines and developed following a feasibility assessment of data availability and completeness, were used to determine when CRFD status was achieved.

**Results:**

Patients (N=333) had a median (interquartile range [IQR]) age of 73.0 (63.0–83.0) years, 58.3% were female and 79.6% were White (Table 2). The median (IQR) duration of IP stay was 6.0 (4.0–9.0) days. Among patients tested, 74.1% (n=229/309) had extended-spectrum β-lactamase-producing Enterobacterales; 78.4% (n=196/250) and 54.7% (n=174/318) had isolates not-susceptible to fluoroquinolones and trimethoprim-sulfamethoxazole, respectively (Table 2). The stringent definition identified 35.1% of patients as CRFD before actual discharge, with a mean [median] difference between CRFD and actual discharge dates of 2.9 [2.0] days (range: 1.0–8.0 days). Using the lenient definition, 57.1% of patients were CRFD by a mean [median] of 3.2 [3.0] days (range: 1.0–10.0 days) before actual discharge.

**Conclusion:**

This study suggests that, even by stringent application of criteria, more than a third of patients with cUTI remained hospitalized longer than clinically necessary, possibly to receive IV carbapenem treatment. These findings emphasize the unmet need for new effective oral antibiotic treatments.

Funding: GSK study 221816.

**Disclosures:**

Serena P. Koenig, MPH, MD, Brigham and Women's Hospital: Employee|GSK: Grant/Research Support Jeffrey J. Ellis, PharmD, MS, GSK: Employee|GSK: Stocks/Bonds (Public Company) Douglas Boettner, PhD, GSK: Employee|GSK: Stocks/Bonds (Public Company) Lindsey Parker, PharmD, GSK: Employee|GSK: Stocks/Bonds (Public Company) Rose Chang, ScD, Analysis Group: Employee|GSK: Grant/Research Support Louise Yu, MS, Analysis Group: Employee|GSK: Grant/Research Support Emily Reichert, MS, Analysis Group, Inc.: Employee|GSK: Grant/Research Support Joanne Chukwueke, MPH, Analysis Group, Inc.: Employee|GSK: Grant/Research Support Zheyi Cao, MS, Analysis Group, Inc.: Employee|GSK: Grant/Research Support Yichuan Grace Hseih, PhD, GSK: Grant/Research Support|Mass General Brigham: Employee Christopher Herrick, MBA, GSK: Grant/Research Support|Mass General Brigham: Employee Mei Sheng Duh, MPH, ScD, Analysis Group: Employee|GSK: Grant/Research Support Shawn N. Murphy, MD, PhD, GSK: Grant/Research Support|Mass General Brigham: Employee

